# Utility of Double Inversion Recovery Sequences in MRI

**DOI:** 10.15844/pedneurbriefs-30-4-1

**Published:** 2016-04

**Authors:** Maura E. Ryan

**Affiliations:** 1 Division of Neuroradiology, Department of Medical Imaging, Ann & Robert H. Lurie Children’s Hospital of Chicago, Chicago, IL; 2 Department of Radiology, Northwestern University Feinberg School of Medicine, Chicago, IL

**Keywords:** Double Inversion Recovery MRI, Focal cortical dysplasia, Epilepsy

## Abstract

Investigators from the Mayo Clinic, Rochester Minnesota investigated the utility of three-dimensional (3D) double inversion recovery (DIR) sequences in magnetic resonance imaging (MRI) detection of focal cortical dysplasia (FCD) in children and young adults with epilepsy.

Investigators from the Mayo Clinic, Rochester Minnesota investigated the utility of three-dimensional (3D) double inversion recovery (DIR) sequences in magnetic resonance imaging (MRI) detection of focal cortical dysplasia (FCD) in children and young adults with epilepsy. Sixteen patients with FCD, 13 patients with periventricular heterotopia and 20 normal patients were evaluated with DIR sequences as well as standard epilepsy MRI 3D magnetization prepared rapid acquired gradient echoes (MPRAGE) sequences. All but one study was performed on a 3 Tesla (T) magnet.

DIR and MPRAGE sequences were independently reviewed by board certified neuroradiologists blinded to the clinical patient data, and scored as normal, indicative of FCD, or periventricular heterotopia. The sensitivity and specificity of the DIR sequence for FCD among all observers ranged from 50-88% and 67-91% respectively, although the consensus among readers with greater experience yielded an 88% sensitivity and 88% specificity for the DIR sequence, compared with a 44% sensitivity and 100 % specificity of for the MPRAGE sequence. The authors concluded that the DIR sequence is sensitive for the detection of focal cortical dysplasia, particularly when reviewed by experienced interpreters, and may be a useful adjunct in MRI imaging of suspected structural epilepsy. [1]

COMMENTARY. FCDs are a possible cause of lesional epilepsy and are potentially curable with resection. Although identification of FCDs by imaging is associated with better post-surgical outcomes, some types of FCDs may be exceedingly subtle or undetectable by MRI, even with dedicated high resolution sequences on high field MRI [2].

Inversion recovery is a MRI technique used to suppress signal from specific tissue or fluid types. DIR sequences suppress both cerebrospinal fluid (CSF) signal and normal white matter signal. This may make abnormal white matter associated with FCDs, such as signal changes at the gray-white junction and radial bands extending toward the ventricular margin, more apparent.

Other studies have also shown that some FCD lesions may be better seen with DIR sequences than with standard MRI sequences such as T2-weighted fluid attenuation inversion recovery (FLAIR) images, which suppress only CSF signal, and 3D MPRAGE [3, 4]. DIR sequences have also been shown to improve detection of other parenchymal changes such as demyelinating plaques of multiple sclerosis [5].

However, it should be noted that DIR sequences also require a relatively longer acquisition time, are prone to CSF pulsation and vascular flow artifact, and interpretation is partially dependent on the experience of the reader. Artifacts likely account for the observed lower specificity of DIR sequences for FCD. Additionally, this sequence has a relatively low signal to noise ratio, which likely reduces the sensitivity at lower, standard 1.5T, magnet strength [5]. Still, preliminary data suggests DIR imaging may be a useful compliment to traditional 3T MRI sequences in patients with epilepsy where subtle FCDs are clinically suspected.
